# Effect of Partial Crystallization on the Structural and Luminescence Properties of Er^3+^-Doped Phosphate Glasses

**DOI:** 10.3390/ma10050473

**Published:** 2017-04-28

**Authors:** Pablo Lopez-Iscoa, Turkka Salminen, Teemu Hakkarainen, Laeticia Petit, Davide Janner, Nadia G. Boetti, Mika Lastusaari, Diego Pugliese, Petriina Paturi, Daniel Milanese

**Affiliations:** 1 Politecnico di Torino, Dipartimento di Scienza Applicata e Tecnologia (DISAT) and INSTM UdR Torino Politecnico, Corso Duca degli Abruzzi 24, Torino 10129, Italy; pablo.lopeziscoa@polito.it (P.L.-I.); davide.janner@polito.it (D.J.); diego.pugliese@polito.it (D.P.); 2 Laboratory of Photonics, Tampere University of Technology, Korkeakoulunkatu 3, 33720 Tampere, Finland; turkka.salminen@tut.fi; 3 Optoelectronics Research Centre, Tampere University of Technology, Korkakoulunkatu 3, 33720 Tampere, Finland; teemu.hakkarainen@tut.fi; 4 nLIGHT Corporation, Sorronrinne 9, 08500 Lohja, Finland; laeticia.petit@tut.fi; 5 Istituto Superiore Mario Boella, Via P. C. Boggio 61, 10134 Torino, Italy; boetti@ismb.it; 6 Department of Chemistry, University of Turku, 20014 Turku, Finland; mika.lastusaari@utu.fi; 7 Turku University Centre for Materials and Surfaces (MatSurf), 20014 Turku, Finland; 8 Department of Physics and Astronomy, Wihuri Physical Laboratory, University of Turku, 20014 Turku, Finland; petriina.paturi@utu.fi; 9 IFN-CNR, CSMFO Lab., Via alla Cascata 56/C, 38123 Povo (TN), Italy

**Keywords:** phosphate glass, glass ceramic, nucleation and growth, Er^3+^ luminescence property

## Abstract

Er-doped phosphate glass ceramics were fabricated by melt-quenching technique followed by a heat treatment. The effect of the crystallization on the structural and luminescence properties of phosphate glasses containing Al_2_O_3_, TiO_2_, and ZnO was investigated. The morphological and structural properties of the glass ceramics were characterized by Field Emission-Scanning Electron Microscopy (FE-SEM), X-ray Diffraction (XRD), and micro-Raman spectroscopy. Additionally, the luminescence spectra and the lifetime values were measured in order to study the influence of the crystallization on the spectroscopic properties of the glasses. The volume ratio between the crystal and the glassy phases increased along with the duration of the heat treatment. The crystallization of the glass ceramics was confirmed by the presence of sharp peaks in the XRD patterns and different crystal phases were identified depending on the glass composition. Sr(PO_3_)_2_ crystals were found to precipitate in all the investigated glasses. As evidenced by the spectroscopic properties, the site of the Er^3+^ ions was not strongly affected by the heat treatment except for the fully crystallized glass ceramic which does not contain Al_2_O_3_, TiO_2_, and ZnO. An increase of the lifetime was also observed after the heat treatment of this glass. Therefore, we suspect that the Er^3+^ ions are incorporated in the precipitated crystals only in this glass ceramic.

## 1. Introduction

Phosphate glasses are of great interest for the manufacturing of photonic devices because of their good chemical stability, easy processing, high rare-earth solubility and excellent optical characteristics [1,2,3,4,5,6]. Due to these properties, phosphate glasses containing rare-earth (RE) ions are very attractive for optical communications [7], laser sources, as well as optical fiber amplifiers [8,9,10]. Interestingly, in the development of these optical devices, the local environment around the RE ions was found to be of paramount importance [11]. Within this framework, different approaches have been pursued to improve the spectroscopic properties of RE-doped glasses. For instance, nanocrystal and ceramic nanoparticle doping methods have been studied in several glass hosts, mainly focusing on silica [12,13,14]. One of the main advantages exhibited by phosphate glasses with respect to the silica counterpart consists in their ability to incorporate higher doping concentrations of erbium ions within the glass matrix.

Glass ceramics (GCs), since their discovery by Stookey in 1953 [15], have been studied as promising materials for applications in electro-optical technology, construction, as well as in dental and bioactive fields [16]. These materials can be obtained by a post-heat treatment of the glass, which promotes in-situ crystal formation in the glass matrix [17]. Thus, GCs combine the glass properties mentioned above with some advantages typical of RE-doped single crystals, such as high absorption/emission and long lifetimes [18]. Moreover, the GCs possess excellent mechanical and thermal durability [19].

Recently, GCs have been further investigated because their luminescence properties may be increased by the crystalline environment around the RE ions [20,21,22,23]. Yu et al. [24,25] reported an increase of about two orders of magnitude in the emission intensity of the Er^3+^/Yb^3+^ co-doped phosphate GCs compared to the correspondent as prepared glasses. Similar results were found by Ming et al. [26].

The addition of metal oxides to phosphate based glasses was found to change their structure, thus modifying the chemical durability, biocompatibility and other properties [27,28,29,30,31,32]. Recently, we reported the effect of the addition of Al_2_O_3_, TiO_2_ and ZnO on the thermal, structural and luminescence properties of Er^3+^-doped phosphate glasses [33]; we found that the addition of Al_2_O_3_ and TiO_2_ promoted connections in the phosphate network, while ZnO acted as a modifier depolymerizing the network.

The present article aims to investigate the impact of the glass composition on the glass crystallization tendency and also the effect of nucleation and growth on the structural and luminescence properties of Er^3+^-doped phosphate glasses. Due to the possibility of increasing the luminescence properties, such as emission and lifetime values, of these glasses, we think that the results of this work will pave the way towards the development of novel promising materials for photonic applications.

## 2. Results and Discussion

**Figure 1:**
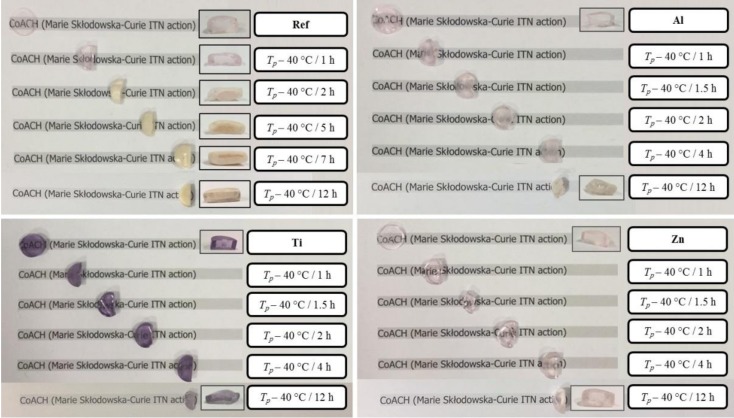
Pictures of the glasses prior to and after different post-heat treatments.

Pictures of the as prepared glasses and of the glasses after different heat treatments are reported in Figure 1: no visible sign of crystallization could be observed in any of the investigated glasses when heat treated at their respective *T_p_* − 40 °C for 1 h. The reference glass ceramic (RefGC) started to be opaque, which is a clear sign of crystallization, whereas the other glasses were still translucent when heat treated at *T_p_* − 40 °C for at least 5 h. A heat treatment at *T_p_* − 40 °C for 12 h led to a complete crystallization only for the RefGC, while all the other glass compositions exhibited a ceramic appearance at the surface due to the surface crystallization and a glassy appearance in the inner part. As shown in Figure 1, the zinc oxide doped glass ceramic (ZnGC) is the least prone to crystallization as compared to the other glasses. The difference in the crystallization tendency of the glasses can be related to their different structures; as explained in [33], Zn ions act as modifiers leading to a depolymerization of the network, which most likely reduces the amount of nucleation sites and therefore decreases the crystallization tendency of the glass. The increase in the glass connectivity induced by the formation of P-O-Al/Ti bonds also seems to delay the crystallization in the aluminum oxide doped glass ceramic (AlGC) and the titanium oxide doped glass ceramic (TiGC). Therefore, the addition of the metal oxides such as Al_2_O_3_, TiO_2_ and ZnO in low concentration (~1.5 mol %) is responsible for the decrease of the glass crystallization tendency.

**Figure 2:**
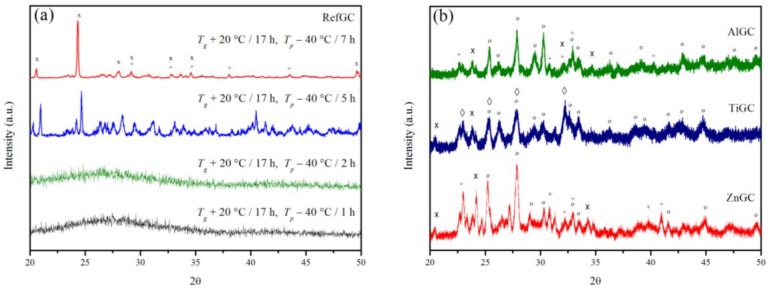
X-ray Diffraction (XRD) patterns of the RefGC after different post-heat treatments (**a**) and of AlGC, TiGC and ZnGC after the heat treatments at *T_g_* + 20 °C for 17 h and *T_p_* − 40 °C for 12 h (**b**). The following crystalline phases were identified: x Sr(PO_3_)_2_ [00-044-0323], + NaSrPO_4_ [00-033-1282], o Sr_3_P_4_O_13_ [04-015-2023] and ◊ Ti(P_2_O_7_) [04-012-4504].

Figure 2a shows the X-ray Diffraction (XRD) patterns of the RefGC after different post-heat treatments at *T_p_* − 40 °C. As can be observed in the figure, the intensity of the peaks rises when the duration of the heat treatment at *T_p_* − 40 °C increases from 1 to 7 h, thus confirming that a longer heat treatment induces the crystal growth. The XRD patterns exhibit sharp peaks after the heat treatment at *T_p_* − 40 °C for 5 and 7 h. Most of the peaks are primarily related to Sr(PO_3_)_2_ [00-044-0323], while the other ones refer to the crystalline phase NaSrPO_4_ [00-033-1282]. Similar crystals were found to precipitate in glasses with similar compositions [34]. The XRD patterns of the other GCs heat treated at *T_p_* − 40 °C for 12 h are presented in Figure 2b. The patterns of the AlGC, TiGC and ZnGC exhibit the same sharp peaks related to Sr(PO_3_)_2_ [00-044-0323] and NaSrPO_4_ [00-033-1282] and also additional peaks which seem to be attributed to Sr_3_P_4_O_13_ [04-015-2023]. A new phase identified as Ti(P_2_O_7_) [04-012-4504] was also detected in the TiGC. It is interesting to point out that none of the crystalline phases contain Er^3+^ ions in their structure.

**Figure 3:**
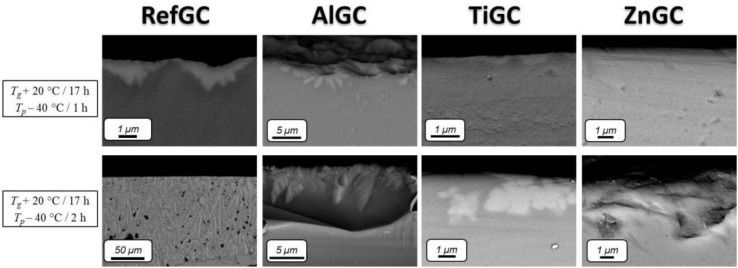
Field Emission-Scanning Electron Microscopy (FE-SEM) images of the cross-section of the glass ceramics (GCs) after the heat treatment at *T_g_* + 20 °C for 17 h and *T_p_* − 40 °C for 1 and 2 h.

**Figure 4:**
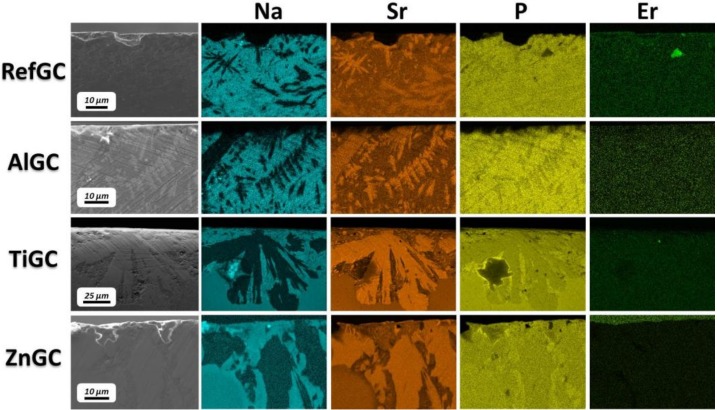
FE-SEM images and Energy Dispersive Spectroscopy (EDS) mapping of the cross-section of the GCs after the heat treatment at *T_g_* + 20 °C for 17 h and *T_p_* − 40 °C for 12 h (brighter areas indicate higher element content).

Figure 3 shows the Field Emission-Scanning Electron Microscopy (FE-SEM) micrographs of the cross-section of the GCs. The external surface of the samples, displayed at the top of the images, highlights evident signs of surface crystallization. The thickness of the crystallized layer rises with an increase in the duration of the post-heat treatment, in agreement with the results reported in [34]. Interestingly, the RefGC exhibits the highest crystallization rate: after 2 h at *T_p_* − 40 °C, large crystals can be evidenced in the RefGC, while crystallization appears to have occurred to a significantly lesser degree in the other GCs. The crystals precipitating in the RefGC and AlGC seem to grow dendritically with split ends, whereas in the case of TiGC and ZnGC the crystals show rounded borders. Additionally, as reported in Figure 4, the Energy Dispersive Spectroscopy (EDS) mapping of the GCs post-heat treated at *T_p_* − 40 °C for 12 h clearly shows Sr-rich crystals surrounded by Na-rich crystals. The Sr-rich crystals seem to have a lower P content than the Na-rich ones. The Er^3+^ ions seem to be homogeneously distributed in all the glass ceramics, but their concentration is probably too small to show clear variations.

**Figure 5:**
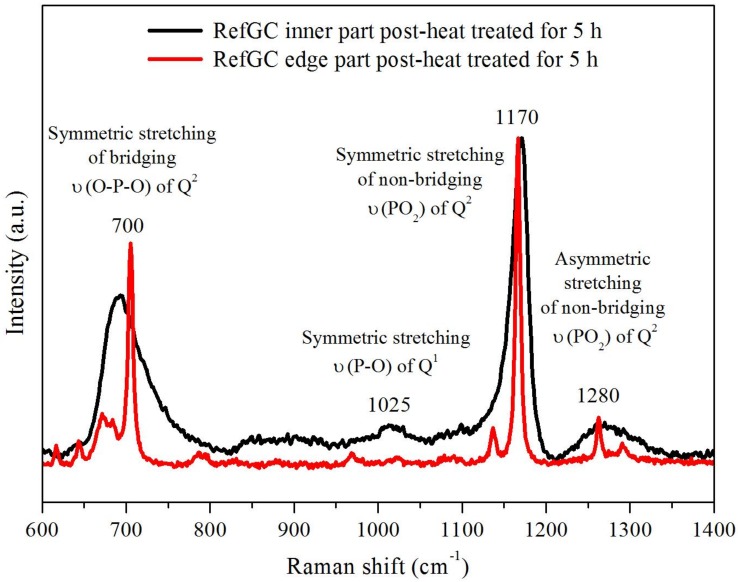
Micro-Raman spectra of the inner and edge parts of the RefGC post-heat treated at *T_p_* − 40 °C for 5 h.

The micro-Raman spectra of the edge crystal and inner glassy parts of the RefGC heat treated at *T_p_* − 40 °C for 5 h are shown in Figure 5. The spectra were normalized at the maximum point (~1170 cm^−1^). The network of a phosphate glass consists of PO_4_ tetrahedral units described using the Q^n^ designation, where n represents the number of bridging oxygens (BOs). The spectrum of the glassy part of the RefGC exhibits defined bands at ~700, 1170 and 1280 cm^−1^ and smaller bands between 800 and 1110 cm^−1^. A complete analysis of the Raman spectrum of the glass can be found in [33]. The bands at around 700 cm^−1^ and at 1025 cm^−1^ correspond to the symmetric stretching bridging ν_sym_(O-P-O) of Q^2^ groups and to the symmetric stretching ν_sym_(P-O) of terminal Q^1^ groups, respectively [35]. The bands at 1170 and 1280 cm^−1^ can be ascribed to the symmetric and asymmetric stretching of non-bridging ν(PO_2_) of Q^2^ groups, respectively [36,37,38]. This spectrum clearly shows the typical structure of a metaphosphate glass [39]. The Raman spectrum measured in the crystal part exhibits a reduction between 15% and 25% of the full width at half maximum (FWHM) of the bands attributed to the Q^2^ groups, confirming the crystallization. Interestingly, the ratio Iν_sym_(P-O-P)/Iν_sym_(PO_2_) between the intensities of the peaks at 700 and 1170 cm^−1^ increases from 0.5 to 0.7 after the heat treatment. At the same time, a depletion of the peak at 1025 cm^−1^ associated with the terminal Q^1^ groups is observed. Thus, these results might be evidence of an increase in the polymerization of the phosphate network after the heat treatment.

**Figure 6:**
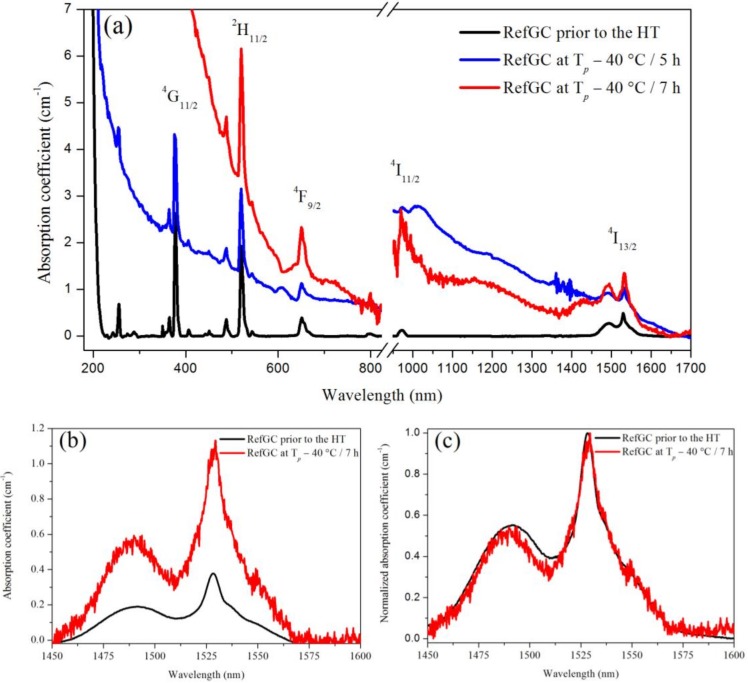
Whole absorption spectra of the RefGC prior to and after the heat treatments (HT) at *T_g_* + 20 °C for 17 h and *T_p_* − 40 °C for 5 and 7 h (**a**); Absorption spectra (**b**) and normalized absorption spectra (**c**) in the range between 1450 and 1600 nm of the RefGC prior to and after the heat treatment at *T_g_* + 20 °C for 17 h and *T_p_* − 40 °C for 7 h.

The absorption spectra of the RefGC prior to and after the heat treatments at *T_g_* + 20 °C for 17 h and *T_p_* − 40 °C for 5 and 7 h are reported in Figure 6a. The heat treatments proved to induce a shift of the absorption edge towards longer wavelengths, which was also evident in all the other glass ceramics (data not shown). A zoom-up of the spectra in the range between 1450 and 1600 nm is shown in Figure 6b. An increase in the absorption coefficient at ~1532 nm can be observed after the heat treatment of the RefGC. Similar results were obtained for the other GCs (data not shown). This might be related to the presence of crystals with a large size. According to the scattering theory and to [40], big crystals produce differences between the refractive index of the crystal and the glassy parts, which provide high scattering. Thus, the opacity of the GCs as well as their absorption coefficient increase along with the crystal size. No significant variations in the shape of the normalized absorption band can be observed in Figure 6c. This might indicate that the heat treatment and thus the formation of crystals do not influence the site of the Er^3+^ ions.

**Figure 7:**
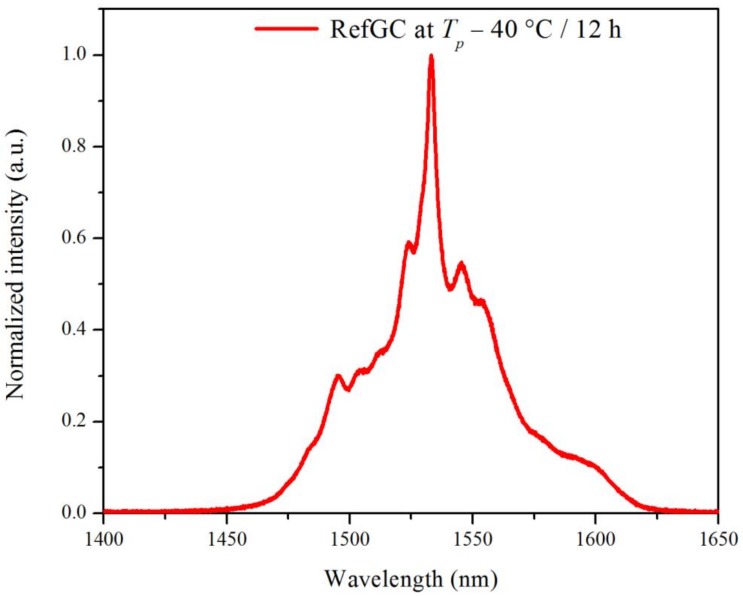
Normalized emission spectrum of the RefGC after the heat treatment at *T_g_* + 20 °C for 17 h and *T_p_* − 40 °C for 12 h.

The emission spectra of the glass ceramics after the heat treatment were measured under excitation at 980 nm. As an example, Figure 7 depicts the normalized emission spectrum of the RefGC after the heat treatment at *T_g_* + 20 °C for 17 h and *T_p_* − 40 °C for 12 h. The spectrum exhibits the typical emission band assigned to the Er^3+^ transition from ^4^I_13/2_ to ^4^I_15/2_. The heat treatment was responsible for a noticeable change in the shape of this emission band only for the RefGC, while its effect can be considered negligible for all the other GCs (data not shown), in agreement with no change in their lifetime values (see Table 1).

Fluorescence lifetime values of the Er^3+^:^4^I_13/2_ level upon 976 nm excitation for all the GCs manufactured are listed in Table 1. An increase in the duration of the heat treatment at *T_p_* − 40 °C leads to an increase of the Er^3+^:^4^I_13/2_ lifetime in the RefGC, while no changes in the lifetime values were observed for the AlGC, TiGC and ZnGC. The increase in the lifetime values recorded for the RefGC sample is attributable to a change in the local environment surrounding the Er^3+^ ions. This could be either associated with their incorporation inside the crystalline phases or with an increase in the interionic distance within the amorphous matrix, both of which are capable of reducing multiphonon decay or clustering effects.

## 3. Materials and Methods

### 3.1. Glass Ceramics Preparation

Glass ceramics with compositions [0.25 Er_2_O_3_ − (0.5 P_2_O_5_ − 0.4 SrO − 0.1 Na_2_O)_100-x_ − (TiO_2_/Al_2_O_3_/ZnO)_x_], with x = 0 and x = 1.5 mol%, were synthesized. The GCs with 1.5 mol% Al_2_O_3,_ TiO_2_ and ZnO are labeled as AlGC, TiGC and ZnGC, respectively, while the glass ceramic with x = 0 is labeled as RefGC. The GCs were obtained by growing in-situ the crystals in the glass matrix.

The glasses were prepared by the conventional melt-quenching technique using NaPO_3_ (Alfa Aesar, Haverhill, MA, USA), SrCO_3_ (Sigma-Aldrich, Saint Louis, MO, USA, ≥99.9%), Er_2_O_3_ (MV Laboratories Inc., Frenchtown, NJ, USA, 99.999%), Al_2_O_3_ (Sigma-Aldrich, Saint Louis, MO, USA, ≥99.5% α-phase), TiO_2_ (Sigma-Aldrich, Saint Louis, MO, USA, 99.99% rutile) and ZnO (Sigma-Aldrich, Saint Louis, MO, USA, ≥99%). Sr(PO_3_)_2_ precursor was independently prepared using SrCO_3_ and (NH_4_)_2_HPO_4_ as raw materials and heating them up to 850 °C. The chemicals were ground and mixed to prepare a 40 g batch, then placed in a quartz crucible and heated up to 1100 °C for 30 min with a heating rate of 10 °C/min. The melt was poured into a preheated brass mold and annealed at 400 °C for 5 h in order to release the residual stress. Finally, the glasses were cooled down to room temperature.

Afterwards, the as-prepared glasses were cut into disks of 10 mm width and 5 mm thickness and were post-heat treated with a heating ramp of 20 °C/min at *T_g_* + 20 °C for 17 h to start nucleation and at *T_p_* − 40 °C from 1 to 12 h to grow the nuclei into the crystals. For the heat treatment, the glass disks were placed on a platinum foil to prevent any contamination from the sample holder and the heat treatments were performed in air. The temperature and duration of the heat treatments were selected on the basis of a previous study on the crystallization behavior of glasses with similar compositions [34]. Finally, the GCs were ground or the cross-sections of the GCs disks were optically polished, depending on the characterization technique.

### 3.2. Glass Ceramics Characterization

The composition and morphology of the samples were determined using a Field Emission-Scanning Electron Microscope (FE-SEM, Zeiss Merlin 4248, Oberkochen, Germany) equipped with an Oxford Instruments X-ACT detector and Energy Dispersive Spectroscopy Systems (EDS/EDX). The formation of crystals at the surface after the post-heat treatment was evidenced by FE-SEM/EDS mapping (Carl Zeiss Crossbeam 540 equipped with Oxford Instruments X-Max^N^ 80 EDS detector). The images were taken at the cross-section of the GCs, previously cut and optically polished. The samples were coated with a thin carbon layer before the EDS mapping.

Raman spectra were acquired with a Renishaw inVia Reflex micro-Raman spectrophotometer (Renishaw plc, Wotton-under-Edge, UK) equipped with a cooled charge coupled device (CCD) camera using a 785 nm excitation line.

The crystalline phases were identified using an X-ray Diffraction (XRD) analyzer (Philips X’pert) with Cu K_α_ X-ray radiation (λ = 1.5418 Å). Data were collected from 2θ = 0 up to 60° with a step size of 0.003°.

The absorption spectra were measured at room temperature using an Ultraviolet-Visible-Near Infrared (UV-Vis-NIR) Agilent Cary 5000 spectrophotometer (Agilent, Santa Clara, CA, USA). The spectra were corrected for Fresnel loss and sample thickness. The samples used for the absorption measurements were GC cross-section disks of 1 mm thickness and optically polished.

The micro-photoluminescence (micro-PL) spectra were measured at room temperature using a continuous wave 980 nm laser diode for excitation. The excitation laser beam was focused on the sample with a 40× magnifying high-numerical aperture (NA) objective. The photoluminescence signal was collected with the same objective and detected with an Andor Idus InGaAs detector attached to a 750 mm Andor Shamrock 750 spectrometer with 1200 l/mm grating. The spatial resolution of the micro-PL setup was 1 µm. 

The fluorescence lifetime of Er^3+^:^4^I_13/2_ energy level was obtained by exciting the samples with a fiber pigtailed laser diode operating at the wavelength of 976 nm, recording the signal using a digital oscilloscope (Tektronix TDS350) and fitting the decay traces by single exponential. All lifetime measurements were collected by exciting the samples at their very edge to minimize reabsorption. Estimated error of the measurement was ± 0.20 ms. The detector used for this measurement was a Thorlabs PDA10CS-EC. The samples used for the emission and lifetime measurements were disks with a thickness of 5 mm.

## 4. Conclusions

GCs in the system P_2_O_5_-SrO-Na_2_O were successfully fabricated by melt-quenching technique followed by a post-heat treatment. Different rates of surface crystallization occurred depending on the composition, with the ZnGC being the least crystallized. Based on the XRD and micro-Raman analyses, Sr(PO_3_)_2_ appears to be the main phase of the crystals grown in the Er^3+^-doped GCs. The fully crystallized RefGC displays the longest lifetime. This experimental evidence should be the subject of further investigations, since it is crucial to assess the possible interplay between the rearrangement of the glass network and the solid state diffusion in influencing the changes in the spectroscopic properties. The erbium ions might have experienced variations in the local environment, which should be confirmed through a future study involving advanced characterization techniques. Furthermore, no change both in the erbium ions lifetime values and in the shape of the emission band located at around 1550 nm was observed after the heat treatment for all the other GCs. Noticeable effects due to the thermal treatment could be highlighted by increasing the Al, Ti and Zn content.

## Figures and Tables

**Table 1:** Excited state ^4^I_13/2_ lifetime values of the GCs under laser excitation at 976 nm.

Heat treatment	*τ* (ms) ± 0.20 ms RefGC	*τ* (ms) ± 0.20 ms AlGC	*τ* (ms) ± 0.20 ms TiGC	*τ* (ms) ± 0.20 ms ZnGC
*T_p_* − 40 °C / 1 h	4.23	4.57	3.73	4.72
*T_p_* − 40 °C / 2 h	4.35	4.54	3.74	4.70
*T_p_* − 40 °C / 5 h	4.43	-	-	-
*T_p_* − 40 °C / 7 h	4.80	-	-	-
*T_p_* − 40 °C / 12 h	6.16	4.46	3.70	4.62
